# Investigating the Post-Mortem Risk of Transmission of SARS-CoV-2 Virus in Cadaveric Tissues: A Systematic Review of the Literature

**DOI:** 10.3390/microorganisms13020284

**Published:** 2025-01-27

**Authors:** Matteo Antonio Sacco, Saverio Gualtieri, Aurora Princi, Maria Cristina Verrina, Angela Carbone, Lucia Tarda, Francesco Ranno, Santo Gratteri, Isabella Aquila

**Affiliations:** Institute of Legal Medicine, Department of Medical and Surgical Sciences, “Magna Graecia” University, 88100 Catanzaro, Italy; matteoantoniosacco@gmail.com (M.A.S.); saveriogualtieri@icloud.com (S.G.); aurora.princi@studenti.unicz.it (A.P.); mariacristina.verrina@studenti.unicz.it (M.C.V.); angela.carbone1@studenti.unicz.it (A.C.); lucia.tarda@studenti.unicz.it (L.T.); francesco.ranno@studenti.unicz.it (F.R.); gratteri@unicz.it (S.G.)

**Keywords:** COVID-19, SARS-CoV-2, cadaver

## Abstract

The emergence of SARS-CoV-2, responsible for the COVID-19 pandemic, has prompted extensive research into its transmission dynamics; yet, a critical aspect that remains underexplored is the post-mortem infectivity of the virus within cadaveric tissues. Understanding the mechanisms by which SARS-CoV-2 maintains infectivity after death is essential, as it raises significant concerns regarding public health and forensic practices. Research indicates that the virus can persist in various tissues, including lung, liver, and kidney tissues, with studies showing that factors such as the time elapsed since death, the presence of underlying health conditions, and environmental conditions at the time of death can influence the level of infectivity in deceased individuals. These findings are not only crucial for establishing safety protocols for forensic investigators who handle cadavers but also for informing public health guidelines that govern the management of bodies during and after outbreaks. As we investigate the implications of post-mortem SARS-CoV-2 infectivity, it becomes imperative to establish comprehensive protocols to mitigate risks associated with the handling and disposal of infected bodies, thereby protecting public health and ensuring the safety of those working in forensic environments. This paper aims to elucidate the mechanisms of infectivity in cadaveric tissues, explore the persistence of the virus in various tissue types, and assess the broader implications for public health and forensic investigations, ultimately contributing to a safer approach in dealing with COVID-19-related fatalities.

## 1. Introduction

The SARS-CoV-2 virus, responsible for the COVID-19 pandemic, exhibits a highly intricate genetic makeup that is vital to its function and pathogenicity. This virus belongs to the beta coronavirus lineage B and features a spherical shape, enclosing a single-stranded, positive-sense RNA genome [1]. The genome’s size, ranging from 27 to 32 kilobases, makes it the largest among RNA viruses, which is significant as it contains the nucleocapsid protein (N) that plays a crucial role in the packaging of this genetic material [2]. The genomic RNA is non-segmented and capped at the 5′ end with a poly-A tail at the 3′ end, facilitating its replication and translation in host cells [3]. Within this genome, approximately 7096 residue-long polyproteins are encoded, comprising both structural and non-structural proteins [4]. These components orchestrate a complex interplay that allows the virus to replicate and evade host immune responses effectively. In addition to structural proteins, SARS-CoV-2 encodes several non-structural proteins (nsps) that are essential for its replication and ability to evade the host immune response. Among these, Mpro (main protease) and PLPro (papain-like protease) play critical roles. Mpro is responsible for processing the polyprotein encoded by the viral genome into functional components necessary for replication and transcription [5]. Similarly, PLPro is involved in processing polyproteins and also acts as a virulence factor by interfering with host immune pathways, such as the ubiquitin and interferon signaling cascades [6]. These proteases are not only vital for the virus’s life cycle but also represent key therapeutic targets for antiviral drug development.

The spike (S) protein is a defining characteristic of the SARS-CoV-2 structure, playing a pivotal role in the virus’s ability to infect host cells. Functionally, the spike protein is a homotrimeric class I fusion protein that extends from the viral surface, enabling the virus to attach to host cells [7]. It is responsible for the virus’s entry into the host by binding to the angiotensin-converting enzyme 2 (ACE2) receptor on the cell surface, a process that is critical for viral pathogenesis [8]. The spike protein consists of two subunits, S1 and S2, which result from cleavage by the enzyme furin during maturation [9]. This cleavage is essential for activating the spike protein’s fusogenic capabilities, facilitating the fusion of the viral and cellular membranes. As the primary target for neutralizing antibodies, the spike protein is a central focus of vaccine and antiviral drug development, underscoring its importance in the immune response and therapeutic interventions [10].

In addition to the spike protein, the membrane (M) and envelope (E) proteins are integral to the structure and function of the SARS-CoV-2 virus. These proteins play crucial roles in the viral life cycle, particularly in the assembly and release of new viral particles. The envelope protein is involved in several processes, including the modulation of the host cell environment to favor viral replication and pathogenesis [11]. It works in concert with the membrane protein to modulate the maturation and retention of the spike protein, ensuring the efficient assembly of virus-like particles [12]. The membrane protein, being the most abundant structural component, provides the virus with its shape and structural integrity, facilitating the budding of viral particles from the host cell [13]. Together, these proteins not only contribute to the virus’s structural stability but also aid in its ability to efficiently propagate and sustain infection within the host.

The process of infection by SARS-CoV-2 involves a coordinated interplay of its structural and non-structural proteins. After the virus enters the host cell via ACE2 receptor-mediated endocytosis, the RNA genome is released and translated into polyproteins. Mpro and PLPro subsequently cleave these polyproteins into functional units, which include the replicase–transcriptase complex (RTC). The RTC facilitates the synthesis of subgenomic RNAs required for the production of structural proteins and the assembly of new virions. Beyond their role in replication, Mpro and PLPro also contribute to the virus’s ability to suppress host immune defenses, underscoring their significance in the viral life cycle and pathogenicity [14].

The mechanism of SARS-CoV-2 infection begins with its entry into human cells, primarily facilitated by the spike (S) proteins on the virus’s surface. These S proteins recognize and bind to the angiotensin-converting enzyme 2 (ACE2) receptors present on the host cell membrane, a critical step for viral entry [15]. The protease TMPRSS2 assists in this process by cleaving the spike protein to enable membrane fusion and viral entry [15]. Once inside, SARS-CoV-2 utilizes its structural and non-structural proteins to hijack the host cell’s machinery for replication. Among these proteins are the S, E, M, and N structural proteins, along with a series of non-structural proteins like nsp1 and nsp2, which play significant roles in RNA processing and replication [2].

Environmental stability plays a crucial role in the persistence and transmission of SARS-CoV-2. The virus’s ability to remain stable in the air and on surfaces significantly influences how efficiently it can spread [4]. Studies have shown that SARS-CoV-2 can maintain stability for extended periods, such as surviving in unventilated environments like buses for up to 30 min [7]. Factors affecting virus stability include the virus concentration and environmental conditions, which can vary greatly across different surfaces [8]. This variability highlights the challenges in predicting transmission risk based on environmental stability alone. The use of Bayesian regression models has provided insights into the decay rates of SARS-CoV-2 in aerosols and on surfaces, offering valuable data for public health interventions [9].

Host susceptibility to SARS-CoV-2 infection is influenced by a combination of genetic, age-related, and health-related factors. For instance, specific ABO blood groups have been linked to varying levels of susceptibility, with blood group A individuals potentially at higher risk [10]. The persistence of SARS-CoV-2 on environmental surfaces is influenced by several factors that contribute to its stability and resistance. Key variables affecting virus stability include temperature, relative humidity, and initial virus titer [8]. Studies have shown that the virus can remain viable on different surfaces for varying durations, ranging from hours to several days [11]. Cold-chain environments, which provide favorable conditions for virus stability, highlight the importance of understanding these factors to develop effective disinfection measures [12]. Additionally, stability in the air and on surfaces significantly impacts the efficiency of SARS-CoV-2 transmission [13]. While sunlight has been shown to rapidly inactivate the virus on surfaces, reducing persistence and exposure risk, these environmental factors remain a critical aspect of understanding the virus’s resistance and transmission dynamics [16]. The infectivity of SARS-CoV-2 in post-mortem settings is an area of growing concern, particularly for healthcare workers, forensic scientists, and other personnel involved in the handling of deceased individuals infected with the virus. Understanding the persistence of SARS-CoV-2 in cadavers is critical for assessing the potential risks of transmission during autopsy procedures or other post-mortem activities. Moreover, this knowledge can inform the development of evidence-based guidelines for the safe handling, transportation, and disposal of bodies, thereby reducing the risk of secondary infections. Investigating the virus’s stability in different tissues and environmental conditions can also shed light on the mechanisms underlying its persistence, providing valuable insights for both public health interventions and virological studies.

The environmental stability of SARS-CoV-2, coupled with its potential to persist in biological tissues post-mortem, highlights the importance of comprehensive studies in this domain. Previous research has demonstrated that SARS-CoV-2 can remain viable on surfaces and in aerosols for varying durations, influenced by factors such as temperature, humidity, and material type [17]. However, the extent to which the virus retains infectivity in cadaveric tissues remains poorly understood. This gap in knowledge poses challenges for ensuring the safety of those involved in post-mortem examinations and raises questions about the potential for environmental contamination in morgues and other settings. By examining the stability and infectivity of the virus in post-mortem contexts, this study aims to address these critical gaps and contribute to the development of robust biosafety measures.

## 2. Materials and Methods

**Figure 1:**
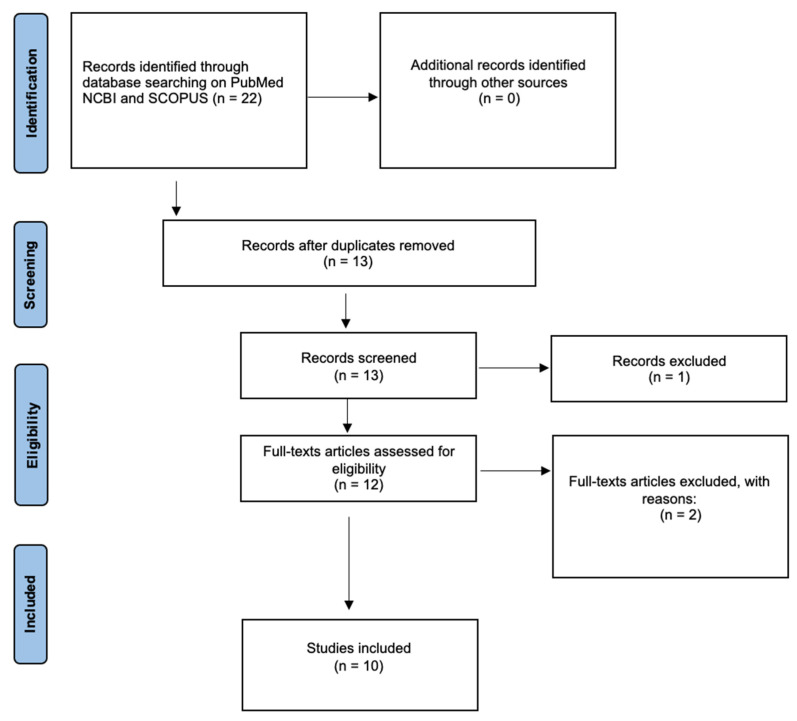
PRISMA flowchart.

We performed a systematic review of the literature on the Pubmed and Scopus search engines. The search was performed according to PRISMA guidelines. The following keywords were used: Covid-19 and cadavers and persistence. Works in English were evaluated. Works that were not related to the topic of the persistence of the virus in cadavers, reviews already carried out, or works for which the full text was not traceable were excluded. The quality control was performed using the Joanna Briggs Institute (JBI, Adelaide, Australia) critical appraisal tools [16] (Figure 1). The review was not registered on standard registration platforms, due to the peculiar topic like the infectivity of the virus in cadaveric tissues. This presents a practical challenge in registering a fully compliant protocol.

## 3. Results

A total of 22 papers emerged from the two search engines. Once duplicates were removed, a total of 13 papers were obtained. Of these, 10 papers were selected according to the inclusion criteria and included in the study.

The results of the individual selected papers are summarized in Table 1.

From the review carried out, it is possible to note in all the cases analyzed the positivity of at least one swab carried out post-mortem to the RT-PCR investigations. Only in three papers were viral cultures performed, of which two papers demonstrated a cytopathic effect.

## 4. Discussion

**Figure 2:**
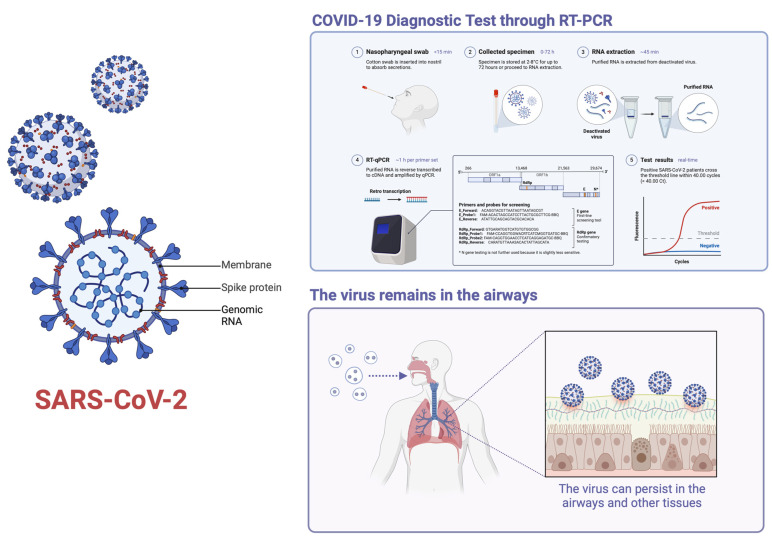
SARS-CoV-2 persistence and diagnostic tests on cadaver (created with BioRender.com; available online (accessed on 18 November 2024)).

The literature review has proved in all the studies described the positivity of at least one of the swabs carried out post-mortem on a corpse, although with different results in the various series and in the various organs. The literature review has demonstrated how SARS-CoV-2 RNA and structural proteins are predominantly found in the respiratory tract of cadavers, particularly in the lungs and pharynx, which are primary sites of infection during life [18,19]. The persistence of RNA in these tissues can be attributed to several factors, including lower temperatures and humidity levels that slow the degradation process, thereby prolonging the post-mortem interval (PMI) of detectable RNA [19,20]. Additionally, the virus’s ability to be detected in organs and tissues of deceased patients who tested positive during their lifetime underscores the challenge in completely eliminating its genetic material post-mortem [2]. Despite the detection of viral components, the infectivity of SARS-CoV-2 in cadaveric tissues seems to be limited even if the risk is not eliminated. The presence of viable virus may persist up to 40 days after death, especially in the lungs, trachea, and perioral areas, but this does not necessarily translate to infectious potential [23]. Studies have shown that SARS-CoV-2 RNA can be detected in lung and heart tissues, with RT-PCR Ct values indicating the presence of viral genetic material even at relatively low concentrations [18,19,20,21,22,23,24,25,26]. This highlights the virus’s remarkable stability and suggests a potential multiorgan distribution due to its tropism for various human organs and tissues. The persistence of viral RNA in different tissues suggests that certain body parts, like the lungs, may serve as niches where the virus can remain undisturbed, even after death (Figure 2).

Differentiating between active and residual SARS-CoV-2 in post-mortem studies presents a unique set of challenges. Post-mortem virology research has highlighted the complexity of distinguishing between active infection and the mere presence of residual viral RNA, a distinction critical for interpreting findings accurately. In particular, in three studies, the effects of the virus were also evaluated by cell culture, with the demonstration of a cytopathic effect in two of these studies [20,25,26]. The persistence of viral RNA in various tissues can result from either ongoing infection or residual shedding, complicating the understanding of the true infectious state of the virus. This differentiation is particularly challenging in individuals who have recovered from COVID-19, as residual viral RNA may still be present long after symptom resolution [11].

The impact of post-mortem viral replication on the persistence of SARS-CoV-2 cannot be underestimated. Although it is commonly believed that viral replication ceases after the host’s death, evidence suggests that SARS-CoV-2 may continue to replicate under certain conditions. For instance, studies have shown that viral RNA can persist and even increase in concentration in specific tissues post-mortem, indicating ongoing replication or stabilization of viral particles [19,26]. This phenomenon could be attributed to the virus’s ability to exploit the residual cellular machinery available in decaying tissues, allowing it to maintain a presence for several days after death [19].

While the immune system is inactive post-mortem, previous immune responses may have induced changes that affect the virus’s ability to persist. For example, the virus may have evolved strategies to evade the host’s immune defenses during active infection, enabling it to remain undetected even after the host’s demise [3]. Additionally, the presence of immune cells and inflammatory markers in tissues could either facilitate or hinder viral persistence. It is possible that these immune remnants create an environment that supports the virus’s survival by providing a niche where it can avoid degradation [19]. Moreover, the extent of tissue damage caused by the immune response during infection may also play a role in determining the virus’s post-mortem survival.

The utilization of PCR in post-mortem analysis has become an essential tool in detecting SARS-CoV-2, providing insight into the virus’s persistence after death. PCR, particularly qRT-PCR, is a highly sensitive method that allows for the detection of SARS-CoV-2 RNA in various tissues, including those preserved in formalin-fixed paraffin-embedded (FFPE) samples or including embalming [25]. Additionally, studies have documented the persistence of SARS-CoV-2 RNA in the upper respiratory tract for extended periods, such as 35 days after death, confirming the robustness of PCR in identifying lingering viral presence [18]. As a primary detection method, PCR not only aids in understanding the virus’s behavior post-mortem but also supports epidemiological studies by providing accurate posthumous infection data.

The level of infectivity in dead bodies is significantly influenced by several factors that present a complex interplay between biological characteristics and procedural management. The risk of transmission during autopsies is notably higher, due to direct contact with biological fluids and tissues and therefore the possibility of operator accidents, thereby necessitating stringent protocols to mitigate the potential exposure of health operators to infectious agents. Furthermore, the presence of SARS-CoV-2 in deceased individuals underscores the importance of conducting thorough histopathological, microbiological, and virologic analyses to gain a comprehensive understanding of the infection dynamics and transmission potential.

The methods used for the preservation of cadavers can significantly influence the persistence of SARS-CoV-2 RNA and its potential infectivity. Studies have shown that refrigeration slows down enzymatic degradation and microbial activity, thereby potentially prolonging the detectability of viral RNA [19,20,21]. Conversely, embalming involves the use of chemical agents, such as formaldehyde, which can inactivate viruses and degrade nucleic acids, reducing the detectability of viral RNA and the viability of infectious particles. For example, Gitto et al. reported a reduction in the abundance of viable SARS-CoV-2 genomes in tissues following embalming procedures [25]. The inclusion of preservation methods in future studies would be critical for a better understanding of how these practices influence viral persistence and inform biosafety protocols.

To better contextualize the findings on SARS-CoV-2, it is valuable to compare its post-mortem behavior with that of earlier coronaviruses, such as SARS-CoV and MERS-CoV. Both SARS-CoV and MERS-CoV have limited evidence in studies about post-mortem transmission during their respective outbreaks, with few conclusive cases reported in forensic or clinical settings [28,29].

In contrast, SARS-CoV-2 has demonstrated remarkable environmental stability and a prolonged ability to persist in tissues post-mortem, as evidenced by the detection of viral RNA and, in some cases, viable virus. This stability is likely influenced by unique structural features, such as the increased efficiency of the spike protein in binding to ACE2 receptors, as well as its broader tropism for various tissues. Furthermore, the global spread of SARS-CoV-2 and the volume of post-mortem cases have increased the likelihood of forensic investigations encountering infected cadavers, necessitating enhanced biosafety protocols.

The longer research timeline for SARS-CoV and MERS-CoV provides a useful comparison, underscoring the unique challenges posed by SARS-CoV-2 in forensic and public health contexts. The distinctive persistence and potential infectivity of SARS-CoV-2 post-mortem highlight the need for tailored approaches in managing cadavers during pandemics.

One important consideration in understanding the post-mortem transmission risk of SARS-CoV-2 is the impact of viral variants. Variants of concern (VOCs), such as Alpha, Delta, and Omicron, have shown significant differences in transmissibility, immune escape, and tissue tropism during life [30]. These characteristics may also influence the persistence and infectivity of the virus in post-mortem settings.

For example, the enhanced binding affinity of the Delta and Omicron variants to ACE2 receptors could hypothetically result in a broader tissue distribution or increased stability in cadaveric tissues. Furthermore, mutations in the spike protein or other genomic regions might affect the virus’s resistance to environmental degradation, potentially altering its post-mortem infectivity.

However, the current literature lacks comprehensive studies directly comparing the behavior of SARS-CoV-2 variants in cadaveric tissues. Preliminary evidence from studies on living patients suggests that variants with higher transmissibility, such as Omicron, may exhibit increased persistence in respiratory secretions [30]. Extrapolating this to post-mortem contexts, variants with higher viral loads during life could maintain detectable RNA levels for longer periods in deceased individuals, raising theoretical concerns about the risk of transmission during autopsy or handling.

To address this gap, future research should prioritize the evaluation of SARS-CoV-2 variants in cadaveric samples, focusing on their persistence, tissue distribution, and infectivity.

Post-mortem infectivity significantly influences public health guidelines, particularly in the context of ensuring safety during necropsy and managing infection risks. The Royal College of Pathologists has emphasized the necessity for updated safety protocols when handling cadavers suspected or confirmed to harbor infectious agents, underscoring the importance of these protocols in mitigating public health risks [31,32]. The relevance of post-mortem microbiological investigations extends beyond individual cases, as these findings can inform public health services about necessary notifications and strategies for infection control, which are pivotal for maintaining public health safety.

In the context of forensic investigations, ensuring the safety of forensic investigations is paramount, given the inherent risks associated with handling biological materials and other potentially hazardous evidence. Investigators must adhere to strict safety protocols to protect themselves and others from exposure to infectious agents, such as those found in post-mortem tissues of individuals who have died from infectious diseases like SARS-CoV-2. These protocols include wearing appropriate personal protective equipment (PPE), such as gloves, masks, and gowns, to minimize the risk of contamination and infection [33]. Furthermore, forensic investigators should collaborate closely with public health officials to stay informed about the latest developments in infectious disease control and integrate these insights into their safety practices.

The detection of the virus in exhumed bodies underscores the potential risk of environmental contamination during corpse treatment processes such as autopsy and embalming, which can introduce contaminants into urban ecosystems. Therefore, even if there is low infectivity, autopsy on COVID-19-positive cadavers is still a procedure with potential occupational risks for operators for which it is essential to adopt prevention and safety measures.

Among the limits of this study, we evidence that one of the inherent challenges in conducting a review on SARS-CoV-2 persistence in cadavers is the limited number of studies currently available on this specific topic. This scarcity may stem from the novelty of the virus, the logistical and ethical complexities of post-mortem studies, and the relatively narrow focus of research in this area. While efforts were made to include all relevant and high-quality studies, the small size of the database inevitably constrains the generalizability and robustness of the conclusions drawn. To mitigate this limitation, we employed a rigorous inclusion and exclusion criteria framework to ensure the reliability and relevance of the selected studies. It is important to note that the limited database highlights the need for further primary research in this field to validate and expand upon the insights provided in this review. Future studies with larger and more diverse datasets will be crucial for a more comprehensive understanding of SARS-CoV-2’s behavior in post-mortem settings.

## 5. Conclusions

The persistence of SARS-CoV-2 in cadavers poses significant challenges for forensic investigations and public health protocols. Current evidence indicates that SARS-CoV-2 RNA and structural proteins can be detected in various tissues and organs, particularly in the lungs, even weeks after death. However, the question of actual infectivity remains largely unresolved. Only a few studies have assessed the virus’s ability to maintain infectious potential through viral cultures, with some demonstrating cytopathic effects. These findings suggest that while viable viral particles may persist, the risk of transmission is likely limited and context-dependent.

The practical implications of these findings are substantial. Stringent safety measures, including the use of personal protective equipment, are crucial during autopsy procedures to minimize risks to healthcare and forensic personnel. Additionally, the need for further research is evident. Future studies should focus on quantitatively evaluating the infectivity of SARS-CoV-2 in different tissues and under various environmental conditions to provide a more comprehensive understanding of the risks associated with post-mortem handling.

Given the limited evidence, this review serves as a starting point for more in-depth investigations and highlights the importance of updating safety protocols for handling cadavers during pandemics and infectious disease outbreaks. Although conclusions about post-mortem transmissibility remain cautious, this work provides a foundation for preventive strategies and future research directions.

## Figures and Tables

**Table 1:** Results of literature review.

Authors	Year	Number of Cases Analyzed	Methods	Sampling Site	Post-Mortem Interval	Results	Geographic Origin
Beltempo et al. [18]	2021	1	RT-PCR	Nasopharyngeal swabs	35 days	Positivity.	Italy
Aquila et al. [19]	2021	29	RT-PCR	Nasopharyngeal or lung swabs	2 h–over 24	10 cases were positive 2 h after death; 10 cases were positive 4 h after death; 9 cases were found positive 6 h after death; 7 cases were positive 12 h after death; 9 cases remained positive 24 h after death.	Italy
Gabbrielli et al. [20]	2021	1	RT-PCR	Lungs and heart	Exhumed (>30 days)	Positivity for some genes.	Italy
			Viral culture			Viral culture negative.	
Sablone et al. [21]	2021	5	RT-PCR	5 μg of tissue biopsy of various organs and swabs of fluids	22–27 days	Positivity for all samples.	Italy
Aiello et al. [22]	2021	14	RT-PCR	Corneal scrapings, conjunctival swabs, and nasopharyngeal swabs	-	NPS and CS were found positive for SARS-CoV-2 RNA in 9/14 (64.3%) and 3/28 (10.7%), respectively. None of the corneal epitheliums showed positivity.	Italy
Bonelli et al. [23]	2022	1	RT-PCR	Nasopharyngeal swabs	Drowned (41 days)	Positivity.	Italy
Sharma et al. [24]	2022	199	RT-PCR	Nasopharyngeal swabs	0–120 h	15 (57.8%) tested positive for COVID-19 after death at different time points, and RT-PCR positivity of SARS-CoV-2 RNA decreases with time.	India
Gitto et al. [25]	2023	4	RT-PCR with viral culture and genome sequencing of SARS-CoV-2	Lungs, liver, spleen, and brain both pre- and post-embalming	2–10 days pre-embalming	All pre-embalming samples were positive, with major material in lungs.	USA
					3–12 days post-embalming	Embalming procedure reduced the abundance of viable COVID-19 genomes in all tissues, even if it was persistent in some lung samples.	
Nagasawa et al. [26]	2024	6	RT-PCR with viral culture and next-generation whole-genome sequencing of SARS-CoV-2	Nasopharyngeal swabs and various tissue samples	4–12 days	RT-PCR positive for all cases. None of the cases showed a clear decreasing trend in viral RNA load.	Japan
						Infectious virus was isolated from Case 1 until 12 days.	
Panda et al. [27]	2024	54	RT-PCR	Skin swab sample from dead bodies and 54 glove samples of handlers were taken within 1 h of death		23/54 (42%) of skin samples were positive;	India
						4/54 (7%) glove samples were positive.	

## References

[1] Rajpal V.R., Sharma S., Sehgal D., Singh A., Kumar A., Vaishnavi S., Tiwari M., Bhalla H., Goel S., Raina S.N. (2022). A comprehensive account of SARS-CoV-2 genome structure, incurred mutations, lineages and COVID-19 vaccination program. Future Virol..

[2] Wang M.Y., Zhao R., Gao L.J., Gao X.F., Wang D.P., Cao J.M. (2020). SARS-CoV-2: Structure, Biology, and Structure-Based Therapeutics Development. Front. Cell Infect. Microbiol..

[3] Wu C.R., Yin W.C., Jiang Y., Xu H.E. (2022). Structure genomics of SARS-CoV-2 and its Omicron variant: Drug design templates for COVID-19. Acta Pharmacol. Sin..

[4] Naqvi A.A.T., Fatima K., Mohammad T., Fatima U., Singh I.K., Singh A., Atif S.M., Hariprasad G., Hasan G.M., Hassan M.I. (2020). Insights into SARS-CoV-2 genome, structure, evolution, pathogenesis and therapies: Structural genomics approach. Biochim. Biophys. Acta Mol. Basis Dis..

[5] Wang Y., Zhou Y., Khan F.I. (2024). Molecular Insights into Structural Dynamics and Binding Interactions of Selected Inhibitors Targeting SARS-CoV-2 Main Protease. Int. J. Mol. Sci..

[6] Ferreira J.C., Villanueva A.J., Al Adem K., Fadl S., Alzyoud L., Ghattas M.A., Rabeh W.M. (2024). Identification of novel allosteric sites of SARS-CoV-2 papain-like protease (PLpro) for the development of COVID-19 antivirals. J. Biol. Chem..

[7] Sternberg A., Naujokat C. (2020). Structural features of coronavirus SARS-CoV-2 spike protein: Targets for vaccination. Life Sci..

[8] Le K., Kannappan S., Kim T., Lee J.H., Lee H.R., Kim K.K. (2023). Structural understanding of SARS-CoV-2 virus entry to host cells. Front. Mol. Biosci..

[9] Bellavite P., Ferraresi A., Isidoro C. (2023). Immune Response and Molecular Mechanisms of Cardiovascular Adverse Effects of Spike Proteins from SARS-CoV-2 and mRNA Vaccines. Biomedicines.

[10] Huang Y., Yang C., Xu X.F., Xu W., Liu S.W. (2020). Structural and functional properties of SARS-CoV-2 spike protein: Potential antivirus drug development for COVID-19. Acta Pharmacol. Sin..

[11] Zhou S., Lv P., Li M., Chen Z., Xin H., Reilly S., Zhang X. (2023). SARS-CoV-2 E protein: Pathogenesis and potential therapeutic development. Biomed. Pharmacother..

[12] Boson B., Legros V., Zhou B., Siret E., Mathieu C., Cosset F.L., Lavillette D., Denolly S. (2021). The SARS-CoV-2 envelope and membrane proteins modulate maturation and retention of the spike protein, allowing assembly of virus-like particles. J. Biol. Chem..

[13] Yang R., Wu S., Wang S., Rubino G., Nickels J.D., Cheng X. (2022). Refinement of SARS-CoV-2 envelope protein structure in a native-like environment by molecular dynamics simulations. Front. Mol. Biosci..

[14] Diogo M.A., Cabral A.G.T., de Oliveira R.B. (2024). Advances in the Search for SARS-CoV-2 M^pro^ and PL^pro^ Inhibitors. Pathogens.

[15] Li J., Jia H., Tian M., Wu N., Yang X., Qi J., Ren W., Li F., Bian H. (2022). SARS-CoV-2 and Emerging Variants: Unmasking Structure, Function, Infection, and Immune Escape Mechanisms. Front. Cell Infect. Microbiol..

[16] Higo A., Palmer S., Liaghat B., Tallis J., Silvester L., Pearce G. (2024). The Effectiveness of Conservative Interventions on Pain, Function, and Quality of Life in Adults with Hypermobile Ehlers-Danlos Syndrome/Hypermobility Spectrum Disorders and Shoulder Symptoms: A Systematic Review. Arch. Rehabil. Res. Clin. Transl..

[17] Salido R.A., Cantú V.J., Clark A.E., Leibel S.L., Foroughishafiei A., Saha A., Hakim A., Nouri A., Lastrella A.L., Castro-Martínez A. (2021). Analysis of SARS-CoV-2 RNA Persistence across Indoor Surface Materials Reveals Best Practices for Environmental Monitoring Programs. mSystems.

[18] Beltempo P., Curti S.M., Maserati R., Gherardi M., Castelli M. (2021). Persistence of SARS-CoV-2 RNA in post-mortem swab 35 days after death: A case report. Forensic Sci. Int..

[19] Aquila I., Ricci P., Bonetta C.F., Sacco M.A., Longhini F., Torti C., Mazzitelli M., Garofalo E., Bruni A., Trecarichi E.M. (2021). Analysis of the persistence time of the SARS-CoV-2 virus in the cadaver and the risk of passing infection to autopsy staff. Med. Leg. J..

[20] Gabbrielli M., Gandolfo C., Anichini G., Candelori T., Benvenuti M., Savellini G.G., Cusi M.G. (2021). How long can SARS-CoV-2 persist in human corpses?. Int. J. Infect. Dis..

[21] Sablone S., Solarino B., Ferorelli D., Benevento M., Chironna M., Loconsole D., Sallustio A., Dell’Erba A., Introna F. (2021). Post-mortem persistence of SARS-CoV-2: A preliminary study. Forensic Sci. Med. Pathol..

[22] Aiello F., Ciotti M., Gallo Afflitto G., Rapanotti M.C., Caggiano B., Treglia M., Grelli S., Bernardini S., Mauriello S., Nucci C. (2021). Post-Mortem RT-PCR Assay for SARS-CoV-2 RNA in COVID-19 Patients’ Corneal Epithelium, Conjunctival and Nasopharyngeal Swabs. J. Clin. Med..

[23] Bonelli M., Rosato E., Locatelli M., Tartaglia A., Falco P., Petrarca C., Potenza F., Damiani V., Mandatori D., De Laurenzi V. (2022). Long persistence of severe acute respiratory syndrome coronavirus 2 swab positivity in a drowned corpse: A case report. J. Med. Case Rep..

[24] Sharma M., Brijwal M., Chakraborty N., Choudhary A., Kumar A., Srivastav S., Lalwani P., Agrawal R., Dev Soni K., Madaan N. (2022). Rate of shed of SARS COV-2 viral RNA from COVID-19 cadavers. J. Infect. Public Health.

[25] Gitto L., Middleton F.A., Reynolds E.S., Thangamani S., Jaeger D.A., Mihaila D.M. (2023). Quantification and persistence of COVID-19 virus in recently deceased individuals before and after embalming. Anat. Sci. Educ..

[26] Nagasawa S., Hirata Y., Miyamoto S., Ozono S., Iida S., Katano H., Tsuneya S., Kira K., Kobayashi S., Nakajima M. (2024). Changes in SARS-CoV-2 viral load and titers over time in SARS-CoV-2-infected human corpses. PLoS ONE.

[27] Panda B., Singh N., Singh G., Patro A.R.K., Mohanty A.P., Patnaik P.K., Misra R. (2024). RT-PCR Result of SARS-CoV-2 Viral RNA in Cadavers and Viral Transmission Risk to Handlers. Indian. J. Crit. Care Med..

[28] Walker D.H. (2016). Value of Autopsy Emphasized in the Case Report of a Single Patient with Middle East Respiratory Syndrome. Am. J. Pathol..

[29] Venkataraman T., Frieman M.B. (2017). The role of epidermal growth factor receptor (EGFR) signaling in SARS coronavirus-induced pulmonary fibrosis. Antivir. Res..

[30] Gomes B.B.M., Ferreira N.N., Garibaldi P.M.M., Dias C.F.S.L., Silva L.N., Almeida M.A.A.L.D.S., de Moraes G.R., Covas D.T., Kashima S., Calado R.T. (2024). Impact of SARS-CoV-2 variants on COVID-19 symptomatology and severity during five waves. Heliyon.

[31] Solarino B., Ferorelli D., Dell’Erba A. (2020). Post-mortem routine practice in the era of the COVID-19 pandemic. J. Forensic Leg. Med..

[32] Tsokos M., Püschel K. (2001). Postmortem bacteriology in forensic pathology: Diagnostic value and interpretation. Leg. Med..

[33] Aquila I., Sacco M.A., Abenavoli L., Malara N., Arena V., Grassi S., Ausania F., Boccuto L., Ricci C., Gratteri S. (2020). Severe Acute Respiratory Syndrome Coronavirus 2 Pandemic. Arch. Pathol. Lab. Med..

